# Sanitary Survey

**Published:** 1848-04

**Authors:** 


SANITARY SURVEYS.

Dr. Edward Jarvis, for some years a citizen of this place, has sent us from Boston a memorial of the American Statistical Association to the Legislature of Massachusetts, praying that honorable body to institute a sanitary survey of the State. The doctor, exceedingly well qualified for the duty, appears as chairman of the committee to which the preparation of the memorial was referred, and we presume it was from his pen. As surveys of this kind are unknown in the West, we give the following extracts from the opening of the memorial, setting forth what the British, French, Belgian and some other European governments have done or are doing on this subject.

"These governments, by especial commissioners or otherwise, have caused sanitary surveys to be made of their respective nations, and thus have learned and published the state of the health and the value of life among the people.

"These sanitary surveys and reports have revealed great differences in the amount of health, strength, and productive power, and in the duration of life, enjoyed by people in different places, and in different classes and conditions of people in the same place; and they have traced and referred many of the diseases to causes which appear to be removable or preventable."

The memorial expresses the opinion "that similar differences of health and longevity may be found in this State, (Massachusetts,) and that many diseases here may likewise be traced to causes that may be removed or prevented."

It contains a variety of interesting facts and views designed to enforce on the Legislature of Massachusetts the propriety of the survey which it proposes.

A tabular view is given of the average duration of life in the different counties of that State, for two years in some and a much longer period in others. The mean term of all the deaths is 32 years 9 months and 28 days—the longest, 40 years and 10 months—the shortest, 22 years 8 months and 19 days. This is in Boston, and shows in a conclusive manner the greater brevity of life in the city than the country. The memorial goes on to say that the Legislature has made geological, trigonometrical and agricultural surveys of the State, has investigated its manufactures, and indirectly, through societies, encouraged farming and improvements of the breeds of domestic animals; when it very properly remarks—

"If these external interests have been deemed worthy of the attention of the Legislature, it would seem that man, the owner and the enjoyer of all these, who is himself a part and parcel of the commonwealth, is much more worthy of legislative interest; and if it is of sufficient consequence to legislate for the growth and strength of cattle, it is of much more consequence to legislate for the health and strength of him for whom alone these creatures are allowed to live. It is not to be expected that any act or power of the government can directly cause a man to be healthy; but it can work as effectually for this as it can to make wheat grow, or an ox become fat. As it has done in regard to animal and vegetable life, the government can search out the circumstances the most favorable to its development and maintenance, and the habits and methods by which it is made most vigorous and enduring."

In reference to this important object the memorial says—

"The first step in this work will be to inquire where and among what classes life is best developed and longest sustained, and where disease is the most prevalent and fatal, and death the most frequent. The next is, to ascertain what are the differences between these places and classes, what differences in the location and what in the circumstances and habits of the people who have such various degrees and amount of life. Having ascertained what and where these injurious causes are, it may then be possible, and perhaps easy, to remove them where they already exist, or to prevent their establishment in places where they have not as yet appeared; and thus the amount of health and the sum of life may be increased among the people."

Our limits do not permit a more extended notice of this appeal to the Legislature. We do not know the result, but sincerely hope it has been, or will be successful, and that Massachusetts will set an example to be speedily followed by other States.

